# Safety and efficacy of a novel ultrathin gastroscope for unsedated transnasal endoscopy in children and adults for evaluation of upper GI disorders

**DOI:** 10.1016/j.igie.2023.12.005

**Published:** 2023-12-23

**Authors:** Aravind Thavamani, Matthew J. Ryan, Kristina Leinwand, Ramya Ramraj, Shauna Schroeder, Paul A. Menard-Katcher, Vrinda Bhardwaj, James P. Franciosi, Joel A. Friedlander, Ramy Sabe

**Affiliations:** 1 Division of Gastroenterology, Hepatology and Nutrition, Department of Pediatrics, University Hospitals Rainbow Babies & Children's, Cleveland, Ohio, USA; 2 Division of Gastroenterology, Hepatology and Nutrition, Department of Pediatrics, The Children's Hospital of Philadelphia, Philadelphia, Pennsylvania, USA; 3 Division of Pediatric Gastroenterology, Department of Pediatrics, Northwest Permanente/Kaiser Permanente, Portland, Oregon, USA; 4 Division of Pediatric Gastroenterology, Department of Pediatrics, Phoenix Children's, Phoenix, Arizona, USA; 5 Division of Gastroenterology and Hepatology, Department of Internal Medicine, University of Colorado School of Medicine - Anschutz Medical Campus, Aurora, Colorado, USA; 6 Department of Pediatrics, Division of Pediatric Gastroenterology, Hepatology and Nutrition Children's Hospital of Los Angeles Los Angeles, California, USA; 7 Division of Gastroenterology, Hepatology and Nutrition, Department of Pediatrics, Nemours Children's Health, Orlando, Florida, USA; 8 EvoEndo, Inc, Centennial, Colorado, USA

## Abstract

**Background and Aims:**

Sedation-free transnasal endoscopy (TNE) is a valuable tool for endoscopic evaluation of the upper GI tract without the risk of general anesthesia. In pediatrics, bronchoscopes are often used for TNE, which precludes gastroduodenal evaluation. We evaluated the use of a novel ultrathin (3.5-mm) gastroscope.

**Methods:**

This multicenter retrospective study involved 7 U.S. hospitals from May 2022 to July 2023. Data were collected from the electronic medical record. The primary outcome was the safety and efficacy measured by completion rate and adverse events. Secondary outcomes were procedure and encounter duration and biopsy sample adequacy.

**Results:**

Fifty-three patients were recruited. Indications were eosinophilic esophagitis surveillance (n = 51) and esophageal variceal evaluation (n = 2). Mean patient age was 15.1 years (range, 6-37 years) with a male predominance (84.9%). Four procedures were unable to be completed. The procedure was successful in 92.4%, and the device success rate was 94.3%. Extent of intended accessibility was transnasal esophagoscopy in 3 patients, transnasal esophagogastroscopy in 42 patients, and transnasal EGD in 4 patients. Almost 40% underwent TNE for the first time. Biopsy samples obtained were adequate for histopathologic analyses. No significant adverse events were observed. Five patients (9.4%) experienced minimal epistaxis, gagging, and nasal pain.

**Conclusions:**

Sedation-free TNE using a single-use gastroscope was well tolerated and safe in pediatric and adult age groups with a success rate similar to previous studies. User feedback noted that the single-use gastroscopes provided improved visualization, increased length, and larger working channel to allow for diagnostic EGD in an efficient ambulatory setting without general anesthesia.

Sedation-free transnasal endoscopy (TNE) is an anesthesia-free means of evaluating the upper GI tract. The flexible endoscopes used in TNE are ultrathin bronchoscopes and gastroscopes measuring 2.8 to 6 mm in diameter, which limits visualization. Bronchoscopes, which are used in children, have smaller working channels. This prevents the use of larger accessories, and the shorter shaft length of the device limits the reach of the endoscope into the stomach and/or duodenum. Thus, we evaluated the safety and efficacy of a newly released single-use gastroscope with a 110-cm length, 3.5-mm outer diameter, and 2-mm accessory channel (EvoEndo, Inc, Centennial, Colo, USA) for sedation-free TNE procedures in children and adults.

The technique of TNE was originally developed by otolaryngologists for the evaluation of the nasal and postnasal cavities and eventually was expanded by gastroenterologist in 1990s for diagnostic evaluations and interventions of the upper GI tract.^1^^,^^2^ Since then, the use of TNE in children and adults has increased significantly for the evaluation of various GI symptoms and disorders including esophagitis (mainly eosinophilic esophagitis), GERD, gastritis, celiac disease, gastroduodenal ulcers, Barrett’s esophagus, dysphagia, and esophageal strictures.^3^, ^4^, ^5^ TNE is effective in both adult and pediatric patients with a success rate approaching 95%.^4^^,^^6^ TNE is usually done in nonsedated awake patients, thus eliminating the costs and risk of sedation and anesthesia endured during routine EGD. This further translates into less time for procedure preparation, quicker visits, no need for special procedure rooms or operating rooms, and overall lower hospital charges.^5^^,^^7^, ^8^, ^9^

## Methods

This multicenter, retrospective study occurred between May 2022 and July 2023 at 7 participating sites across the United States: Children’s Hospital of Philadelphia, UH Rainbow Babies & Children’s Hospital, Children’s Hospital of Los Angeles, UC Health Digestive Health Center, Phoenix Children’s Hospital, Nemours Children’s Hospital, and Northwest Permanente Physicians and Surgeons. All patients underwent a sedation-free TNE using the EvoEndo gastroscopes (EvoEndo, Inc). The EvoEndo model LE gastroscope includes a 110-cm shaft with 4-way tip steering, 3.5-mm outer diameter, and 2-mm accessory channel. It is powered by a 2-pound, 6 × 8-inch controller.

Electronic medical records were reviewed. Patient demographics, indications, extent reached, prior TNE procedures, endoscopic findings, biopsy sample adequacy, setting of the procedure, topical lidocaine use, duration of the procedure, duration of the visit, and adverse events were collected and reviewed. Institutional review board approval was obtained from all participating institutions, starting with University Hospitals Rainbow Babies & Children’s (STUDY20221203) on November 3, 2022.

This is a descriptive study. Categorical variables are reported as numbers and percentages and continuous variables as mean ± standard deviations for parametric data and median with interquartile range (IQR) for nonparametric data.

## Results

Fifty-three patients underwent TNE using EvoEndo gastroscopes during the study period (Table 1). Mean patient age was 15.1 ± 5.6 years. The youngest patient was 6 years old, and the age range of the cohort was 6 to 37 years. The cohort had a male predominance (84.9%). Approximately 40% of the patients underwent TNE with EvoEndo gastroscopes for the first time. The remaining 60.4% had a previous TNE using conventional bronchoscopes. Almost 36% of the patients underwent TNE in an outpatient clinic setting, whereas close to 64% had their procedures completed in a room prepared for a sedation-free procedure in the endoscopy suite. TNE was done as part of a combined outpatient visit in almost 75% of the patients who underwent TNE in a clinic setting. Indications for TNE included eosinophilic esophagitis and esophageal variceal surveillance.

**Table 1:** Demographics of the study population (n = 53)

Demographic characteristics	Values
Age, y	15.1 ± 5.6
Sex	
Male	45 (84.9)
Female	8 (15.1)
Location	
Clinic setting	19 (35.8)
Endoscopy suite	34 (64.2)
First time experience of transnasal endoscopy	18 (38.3)
Procedure duration, min	9.5 (7-16)
Visit duration, min	33.5 (27.7-39.2)
Procedural success rate, %	92.4
Device success rate, %	94.3
Pooled adverse event rate (gagging, nasal pain, epistaxis), %	9.4

Values are mean ± standard deviation, n (%), or median (interquartile range) unless otherwise defined.

Sedation-free TNE was successfully completed in 92.4% of our study population (49/53) with adequate endoscopic visualization and satisfactory biopsy specimens. By removing the patient whose procedure was not started because of anxiety from the cohort, the completion rate was 94.3% (50/53). Among the 49 completed procedures, the extent of intended accessibility using EvoEndo gastroscopes was as follows: transnasal esophagoscopy (TN-Eso) in 3, transnasal esophagogastroscopy in 42, and transnasal EGD (TN-EGD) in 4 patients. The procedure and visit duration were present for 18 patients in our cohort. Of those available, the median procedure duration was 9.5 minutes (IQR, 7-16) and the median total visit duration was 33.5 minutes (IQR, 27.7-39.2). All subjects had topical analgesia using 2% or 4% lidocaine per each site’s protocol. Virtual reality procedural dissociation and distraction during the procedure was also used as clinically indicated. During these procedures, the mean number of proximal and distal esophageal biopsy samples obtained were 3.5 and 3.6, respectively, and all biopsy samples obtained were satisfactory for histopathologic evaluation.

Of the 4 unsuccessful procedures, 1 patient had vasovagal syncope before introduction of the endoscope, and the procedure was aborted, and 3 patients had severe edema of the nasal turbinates with difficult further advancement of the EvoEndo gastroscopes. Those 3 procedures were eventually completed by switching to a smaller conventional bronchoscope (3.1-mm outer diameter, 1.2-mm accessory channel). Almost 90% of the patients tolerated the procedure with no adverse events. Nonsignificant adverse events of epistaxis were observed in 2 patients, nasal pain was reported in 2 patients, and gagging was observed in 1 patient with an overall adverse event rate of 9.4%.

**Figure 1:**
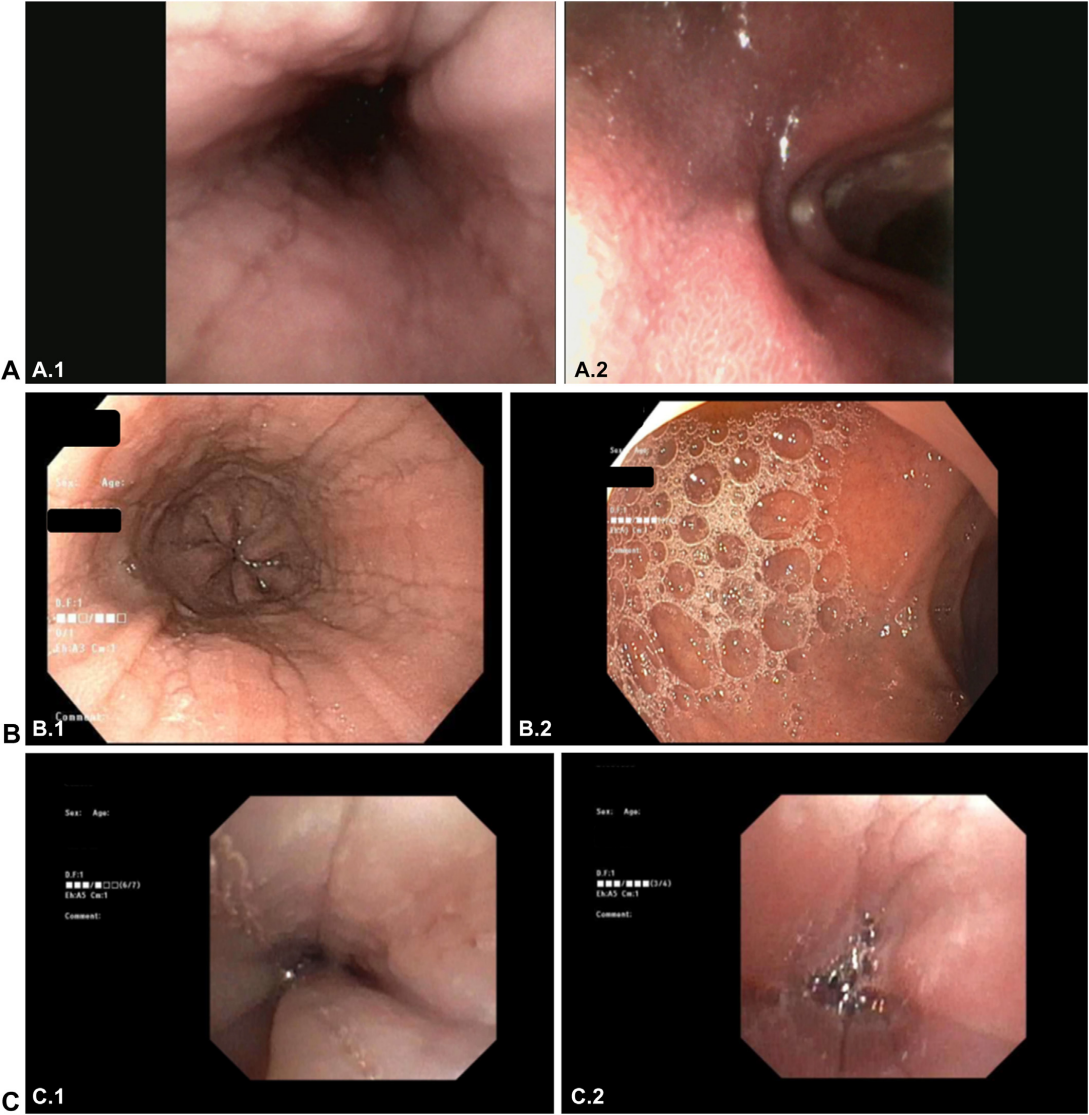
Comparison of picture quality between (**A**) single-use transnasal EvoEndo gastroscopes, (**B**) conventional transoral gastroscopes, and (**C**) conventional bronchoscopes. **A.1,** EvoEndo gastroscope image of esophagus with findings suggestive of eosinophilic esophagitis. **A.2,** EvoEndo gastroscope image of the duodenal bulb with mucosal erythema. **B.1,** Conventional EGD image of the esophagus with findings suggestive of eosinophilic esophagitis. **B.2,** Conventional EGD image of the duodenal bulb without significant visual abnormalities. **C.1** and **C.2,** Conventional bronchoscope images of the esophagus with findings suggestive of eosinophilic esophagitis.

Procedures were completed by 8 pediatric gastroenterologists, 1 pediatric gastroenterology fellow, and 1 adult gastroenterologist. A pediatric otolaryngologist assisted in some procedures at a single institution. The number of TNEs performed by the pediatric and adult gastroenterologists before the time of the study ranged from 23 to 120 TN-Eso and 0 to 8 transnasal esophagogastroscopies. Endoscopists subjectively reported improved visualization and increased functionality with the additional air, suction, and water use and increased length and larger working channel when using the EvoEndo gastroscope for TNE as compared with conventional bronchoscopes. Figure 1 shows the comparison of picture quality between conventional transoral endoscopes and bronchoscopes and single-use EvoEndo gastroscopes.

## Discussion

The advent of small-caliber endoscopes has widened the use of unsedated TN-EGD in both the adult and pediatric populations without any risk of sedation or anesthesia while ensuring high success and tolerance rates, making them an attractive alternative to conventional EGD.^3^^,^^10^ Traditionally, conventional bronchoscopes used for TNE range from 2.8 mm to 4.2 mm in diameter. However, the lack of irrigation features and their small diameter with associated optics can make viewing of mucosa more difficult. This subsequently can hinder physician targeting of biopsy samples from abnormal areas and make calculating endoscopic visualization scores challenging. Furthermore, bronchoscopes (60 cm) are shorter than gastroscopes because they were originally designed for bronchial tree evaluation, which precludes evaluating the stomach and duodenum as part of the TNE procedure. EvoEndo gastroscopes are particularly useful in these settings because they have a similar diameter (3.5 mm) but with superior visualization, and the ability for air, water, and suction use allows providers to better visualize the GI anatomy and to perform targeted interventions. The increased length (110 cm) provides accessibility to the gastric cavity and duodenum for diagnostic and therapeutic interventions to be performed when indicated.

In our study, 18 patients had the procedure duration and total visit duration available. The mean procedure duration was 11 ± 4.7 minutes and the mean total visit duration 33.5 ± 7.2 minutes. The median procedure duration (9.5 minutes [IQR, 7-16]) and median total visit duration (33.5 minutes [IQR, 27.7-39.2]) were a little longer than that reported for TN-Eso in a previous study^8^ (7 minutes [IQR, 6-8.3] and 33.5 minutes [IQR, 29-43], respectively) that used a conventional bronchoscope in children and young adults with eosinophilic esophagitis to evaluate only the esophagus. These differences could be because of the added evaluation of the stomach and/or duodenum in some of our patients, which typically adds to the total duration. This could also be affected by our smaller sample size because data were available for only one-fourth of our patient population because of the retrospective nature of our study. Minor adverse events have been reported in studies evaluating the use of conventional bronchoscopes for TNE including epistaxis, nausea, gagging, vomiting, pain, and tracheal intubation after sneezing in 9.5% to 10.2% of their patients with no reported serious adverse events.^4^^,^^8^ The use of EvoEndo gastroscopes in our study was associated with a similar minor adverse event rate of 9.4% with no serious adverse events.

TNE is overall well accepted and tolerated in both adult and pediatric populations.^7^^,^^10^, ^11^, ^12^ Cho et al^6^ reported that 88% of their adult patients who underwent TN-EGD showed willingness to undergo the procedure again for medical indications. Among patients who already had conventional EGD while under conscious sedation, almost 62% preferred unsedated TN-EGD compared with conventional EGD under sedation. They reported a high degree of acceptability (mean score, 6.6 [range, 1-10]). Friedlander et al^7^ reported similar findings in their study evaluating TN-Eso in children, reporting that all parents and 76.2% of patients would prefer to undergo TN-Eso again. They also reported that 85.7% of parents and 52.4% of patients preferred TN-Eso over sedated EGD, and more than 85% of their patients had tolerated the procedure with ease. The high success and tolerance rates in pediatric and adult patients using conventional bronchoscopes and gastroscopes open the way for evolution in the field of sedation-free TNE. The emergence of a new alternative that provides options to improve tolerance and accessibility enriches the field.

The strength of our study is that it is a multicenter study, providing experience with a novel ultrathin transnasal gastroscope (EvoEndo, Inc). It further pools experiences from different institutions with providers of different TNE experience in patients with diverse demographics. Despite variations in the level of TNE expertise among providers, patient age, and indications for endoscopic evaluation, the overall success rate was high in the pediatric and adult age groups. The limitation of our study is that it is a retrospective study, resulting in inconsistent information collection between all centers including procedure duration and total visit duration. Despite that, the available data are still of value to build on in future studies.

In conclusion, sedation-free TNE is well tolerated and safe in pediatric and adult age groups with a high success rate. EvoEndo gastroscopes provided a safe device alternative with expanded capabilities to allow for a transnasal endoscopic procedure as compared with conventional sedated oral EGD. By removing sedation and having a single-use portable platform, the devices can provide a less costly procedure option for patients and a potentially more prompt yet equivalent diagnostic in-office or clinic evaluation.

## Disclosure

The following author disclosed financial relationships: J. A. Friedlander: Chief Medical Officer, stock holder, and board member of EvoEndo, Inc; He is listed as co-inventor on several patents and patents pending related to endoscopic devices, methods, and virtual reality technologies assigned to the University of Colorado. All other authors disclosed no financial relationships. Research support for this study was provided by EvoEndo, Inc (endoscopes).
